# Inulin Can Alleviate Metabolism Disorders in *ob/ob* Mice by Partially Restoring Leptin-related Pathways Mediated by Gut Microbiota

**DOI:** 10.1016/j.gpb.2019.03.001

**Published:** 2019-04-23

**Authors:** Xiaofeng Song, Liang Zhong, Na Lyu, Fei Liu, Boxing Li, Yanan Hao, Yong Xue, Jing Li, Yuqing Feng, Yue Ma, Yongfei Hu, Baoli Zhu

**Affiliations:** 1 CAS Key Laboratory of Pathogenic Microbiology and Immunology, Institute of Microbiology, Chinese Academy of Sciences, Beijing 100101, China; 2 Savaid Medical School, University of Chinese Academy of Sciences, Beijing 100049, China; 3 Beijing Key Laboratory of Microbial Drug Resistance and Resistome, Beijing 100101, China; 4 Department of Pathogenic Biology, School of Basic Medical Sciences, Southwest Medical University, Luzhou 646000, China; 5 State Key Laboratory of Animal Nutrition, College of Animal Science and Technology, China Agricultural University, Beijing 100193, China; 6 College of Food Science and Nutritional Engineering, China Agricultural University, Beijing 100083, China

**Keywords:** Prebiotics, Gut microbiota, Obesity, Transcriptome, Metabolic disorders

## Abstract

Inulin has been used as a prebiotic to alleviate glucose and lipid metabolism disorders in mice and humans by modulating the **gut microbiota**. However, the mechanism underlying the alleviation of **metabolic disorders** by inulin through interactions between the gut microbiota and host cells is unclear. We use *ob/ob* mice as a model to study the effect of inulin on the cecal microbiota by 16S rRNA gene amplicon sequencing and its interaction with host cells by transcriptomics. The inulin-supplemented diet improved glucose and lipid metabolism disorder parameters in *ob/ob* mice, alleviating fat accumulation and glucose intolerance. The α diversity of gut microbial community of *ob/ob* mice was reduced after inulin treatment, while the β diversity tended to return to the level of wild type mice. Interestingly, *Prevotellaceae UCG 001* (family Prevotellaceae) was obviously enriched after inulin treatment. A comparative analysis of the gene expression profile showed that the cecal **transcriptome** was changed in leptin gene deficiency mice, whereas the inulin-supplemented diet partially reversed the changes in leptin gene-related signaling pathways, especially AMPK signaling pathway, where the levels of gene expression became comparable to those in wild type mice. Further analysis indicated that *Prevotellaceae UCG 001* was positively correlated with the AMPK signaling pathway, which was negatively correlated with markers of glycolipid metabolism disorders. Our results suggest that the inulin-supplemented diet alleviates glucose and lipid metabolism disorders by partially restoring leptin related pathways mediated by gut microbiota.

## Introduction

Inulin is a common prebiotic defined as a non-digestible dietary fiber that supports the growth of probiotics [1]. To be more specific, it can stimulate the growth of bifidobacteria in the intestine [2]. Therefore, inulin has been used to regulate gut microbiota-related disorders, such as metabolic disorders, allergies, and inflammatory bowel disease (IBD) [3], [4], [5], [6]. To date, only one prebiotic has been identified by European Food Safety Authority: inulin improves bowel function [7]. In addition, the probiotic effect of inulin, such as changing the gut microbiota of obese individuals, increasing the abundances of bifidobacteria and *Akkermansia muciniphila* in obese individuals and improving metabolic disorders, have been widely reported [5], [8], [9], [10], [11]. Inulin can be enzymatically hydrolyzed by some bacteria to produce short-chain fatty acids (SCFAs) in the colon, which can bind to G protein-coupled receptors 41/43 (GPR41/43) expressed on the intestinal epithelial cell membrane and then stimulate Glucagon-like peptide-1 (GLP-1) production. As a consequence, GLP-1 has a chronic effect on the host energy metabolism system [12], [13].

Several metabolites produced by the gut microbiota (*e.g.*, SCFAs and lipopolysaccharide (LPS)) can affect host gene expression profile and regulate host energy balance involving free fatty acid receptor (FFA) 2/3, fasting-induced adipocyte factor (Fiaf), and adenosine monophosphate (AMP)-activated protein kinase (AMPK) [13], [14], [15], [16]. Additionally, SCFAs can regulate the secretion of gut peptides (*e.g.*, GLP-1 and Peptides YY (PYY)) by recognizing receptors on the surface of intestinal cells. These gut peptides have positive effects on reducing food intake and improving glucose metabolism [17]. Conversely, LPS, as a type of pathogen-associated molecular patterns (PAMPs), can trigger low-grade inflammation, which leads to the occurrence of metabolism disorders [18].

Obesity is a typical metabolic disorder, and the gut microbiota is regarded as an important body part that may provide additional contributions to obesity besides the contributions of the host genotype and lifestyle. Previous studies have provided solid evidence that the gut microbiota can affect host energy intake [15], [19].

AMPK as a key protein kinases of host energy status, plays an essential role in host energy balance. It can regulate the expression of adipokines, which are involved in body weight control, appetite control, and maintenance of metabolism homeostasis [20]. In general, the role of AMPK in liver, fat, and skeletal muscle tissue is critical, and the activity of AMPK can be tuned by different factors such as drug intervention.

We hypothesize that the AMPK signaling pathway can also play an important role in modulating gene expression of cecal tissue after the inulin-supplemented diet intervention and has effects on other signaling pathways related to obesity. To understand the mechanism underlying the improvement in glycolipid metabolism disorders by inulin in obese individuals, *ob/ob* mice were selected to seek the effect of the inulin-supplemented diet on the cecal microbiota and its possible influence on the host cecal gene expression profile.

## Results

### The inulin-supplemented diet can improve metabolic disorder-related symptoms in genetically obese mice

**Figure 1:**
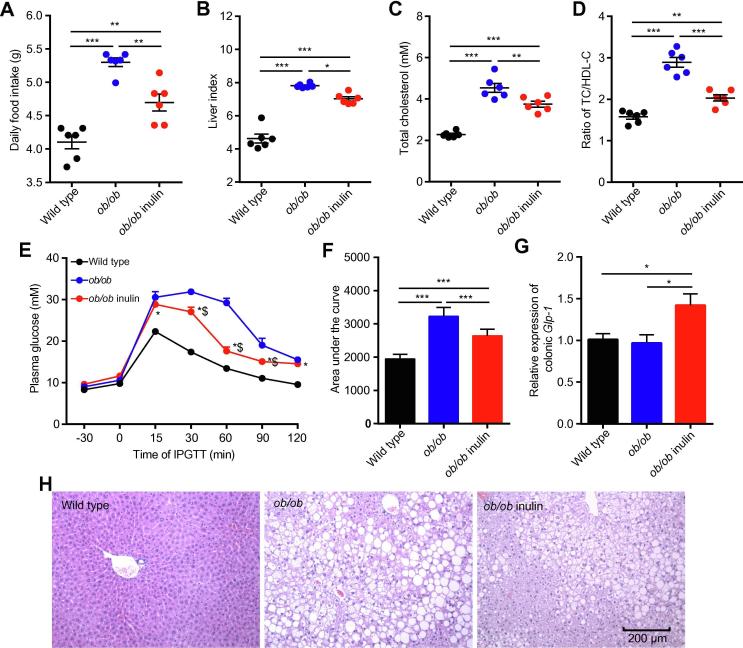
**Improvement in metabolic parameters in *ob/ob* mice by inulin** **A.** Daily food intake. **B.** Liver index. **C.** Serum TC. **D.** Ratio of TC/HDL-C. **E.** Plasma glucose (mM) profile. ^*^P < 0.05 for *ob/ob* inulin versus wild type; ^$^*P* < 0.05 for *ob/ob* inulin versus *ob/ob*. **F.** Mean AUC measured during the IPGTT mM·min). **G.** Relative expression of colonic *Glp-1*. **H.** Representative H&E-stained images of the liver. Scale bars, 200 μm. *n* = 6 per group. TC, total cholesterol; HDL-C, high-density lipoprotein cholesterol; AUC, area under the curve; IPGTT, intraperitoneal glucose tolerance test; *Glp-1*, glucagon-like peptide-1. Data are presented as mean ± SEM. Data were analyzed using one-way ANOVA followed by the Tukey *post hoc* test for A–D, F, and G and with two-way ANOVA followed by the Bonferroni *post hoc* test for E. *n* = 6 per group. ^**^P < 0.01; ^***^P < 0.001.

To investigate the effect of inulin treatment on glycolipid metabolism disorders in genetically obese mice, we examined the body weight, daily food intake, liver weight and glycolipid metabolism related parameters. The initial body weight (Figure S1), daily food intake, liver index, serum total cholesterol (TC), TC/high-density lipoprotein cholesterol (HDL-C) ratio and the area under the curve (AUC) of the Intraperitoneal glucose tolerance test (IPGTT) of the *ob/ob* mice (*ob/ob*) were significantly elevated compared with those of wild type mice (*P* < 0.05, one-way analysis of variance (ANOVA) followed by the Tukey *post hoc* test, Figure 1A–F), indicating severe glucose intolerance and dyslipidemia. In contrast, *ob/ob* mice fed the inulin-supplemented diet (*ob/ob* inulin) for 4 weeks showed improved metabolic parameters. After inulin intervention, the treated group showed no statistically significant difference in body weight change compared with the *ob/ob* group (Figure S2), but had a significantly lower daily food intake (*P* < 0.05, one-way ANOVA followed by the Tukey *post hoc* test, Figure 1A), serum TC, and TC/HDL-C ratio (*P* < 0.05, one-way ANOVA followed by the Tukey *post hoc* test, Figure 1C, D). Additionally, the AUC of IPGTT was lowered, indicating a significant improvement in glucose tolerance (*P* < 0.05, one-way ANOVA followed by the Tukey *post hoc* test, Figure 1E, F). Subsequently, we measured the *Glp-1* mRNA level in colonic tissue. The inulin-supplemented diet could rise the *Glp-1* mRNA level in *ob/ob* inulin, meaning that inulin restored glucose homeostasis in *ob/ob* mice (*P* < 0.05, one-way ANOVA followed by the Tukey *post hoc* test, Figure 1G), which was not reported previously. According to liver tissue staining (Figure 1H), *ob/ob* mice exhibited severe hepatic steatosis, which was alleviated after inulin treatment. Overall, the above results indicate that inulin has a beneficial effect that ameliorates glycolipid metabolism disorders in *ob/ob* mice.

### The *ob/ob* mice fed the inulin-supplemented diet show apparent changes in gut microbial structure

**Figure 2:**
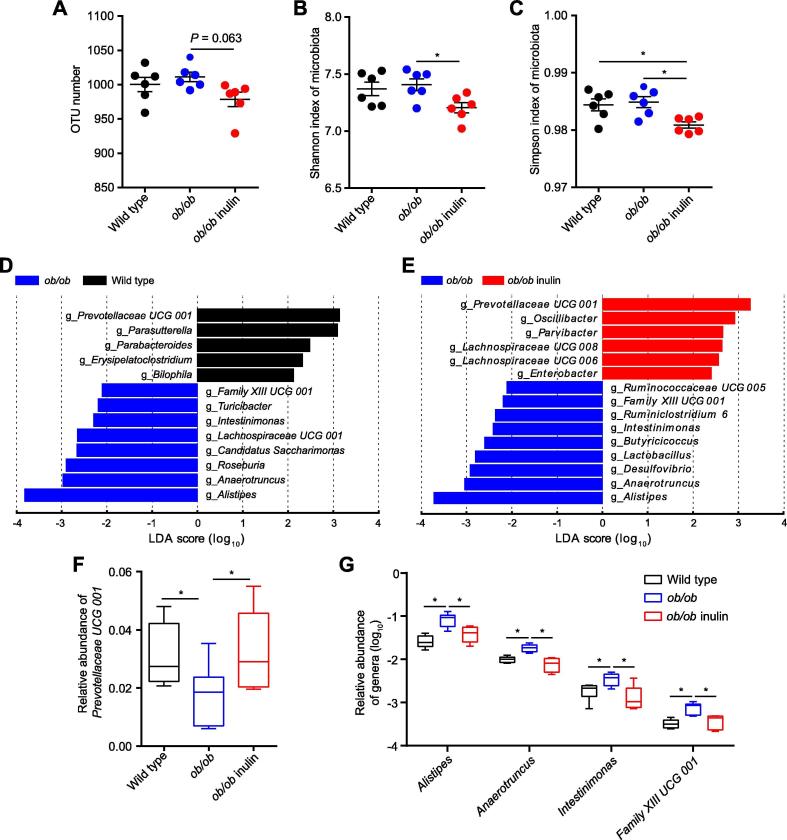
**Inulin modifies the composition of the cecal microbiota in *ob/ob* mice** **A.** OTU number. **B.** Shannon index of microbiota. **C.** Simpson index of microbiota. **D.**. LDA scores of differentially abundant taxa between the wild type and *ob/ob* mice using the LEfSe method. **E.** LDA scores of differentially abundant taxa between the *ob/ob* and *ob/ob* inulin mice using the LEfSe method. **F.** Relative abundance of *Prevotellaceae UCG 001*. **G.** Relative abundance of *Alistipes*, *Anaerotruncus*, *Intestinimonas*, and *Family XIII UCG 001*. OTU, operational taxonomic unit. Data were analyzed using one-way ANOVA followed by the Tukey *post hoc* test for A–C, and Kruskal–Wallis sum-rank test and Wilcoxon rank-sum test for D, E. *n* = 6 per group. ^*^P < 0.05.

To explore the effect of inulin treatment on the gut microbiota of *ob/ob* mice, we performed 16S rRNA sequencing of the cecal contents. We used the 16S rRNA gene amplicon sequencing method (V3-V4 region) and generated 3,179,284 reads for a total of 18 samples, with an average of 176,627 ± 22,171 reads per sample. Compared with the genetically obese mice, the treated group had slightly fewer operational taxonomic units (OTUs) (*P* = 0.063, one-way ANOVA followed by the Tukey *post hoc* test, Figure 2A) and a significantly lower α diversity index (Shannon and Simpson indexes) (*P* < 0.05, one-way ANOVA followed by the Tukey *post hoc* test, Figure 2B, C). The principal coordinates analysis (PCoA) of the weighted UniFrac distances analysis for the gut microbiota of the three mouse groups showed that the wild type and *ob/ob* groups were clearly clustered into two separate groups, while the values for the *ob/ob* inulin mice were clustered between the two groups. However, the three groups shared some overlapping regions (Figure S3). In summary, we did not detect significant differences in bacterial diversity between wild type and *ob/ob* mice, while there was a slightly lower bacterial diversity in *ob/ob* inulin mice.

The phylum Bacteroidetes was dominant among the 9 phyla present in the gut microbiota from the three groups of mice, and the ratio of Firmicutes*/*Bacteroidetes was increased in *ob/ob* mice over wild type group, but lower in the *ob/ob* inulin group compared with *ob/ob* mice (Figure S4). The gut microbiota in obese individuals has usually shown an increased Firmicutes*/*Bacteroidetes ratio [21]. Therefore, the decreased Firmicutes*/*Bacteroidetes ratio of *ob/ob* inulin means that this feature in obesity could be reversed by the inulin-supplemented diet.

Next, to identify the changes in specific bacterial taxa after the inulin-supplemented diet intervention, we utilized the linear discriminant analysis (LDA) effect size (LEfSe) to compare the cecal microbiota composition between the *ob/ob* and *ob/ob* inulin groups. At the genus level, LDA score was selected to discriminate specific taxa in different groups. Compared with the wild type group, the *ob/ob* mice had a higher abundance of *Alistipes*, *Anaerotruncus*, *Roseburia*, *Candidatus Saccharimonas*, *Lachnospiraceae UCG 001*, *Intestinimonas*, *Turicibacter*, and *Family XIII UCG 001* but a lower abundance of *Prevotellaceae UCG 001*, *Parasutterella*, *Parabacteroides*, *Erysipelatoclostridium*, and *Bilophila* (Figure 2D). Correspondingly, *Alistipes*, *Anaerotruncus*, *Desulfovibrio*, *Lactobacillus*, *Butyricicoccus*, *Intestinimonas*, *Ruminiclostridium 6*, *Family XIII UCG 001*, and *Ruminococcaceae UCG 005* were enriched in the *ob/ob* group, and *Prevotellaceae UCG 001*, *Oscillibacter*, *Lachnospiraceae UCG 006*, *Lachnospiraceae UCG 008*, *Enterobacter*, and *Parvibacter* were increased by inulin treatment in the *ob/ob* mice (Figure 2E). From the results of the above LEfSe analyses at the genus level, we obtained 5 genera, *Prevotellaceae UCG 001*, *Alistipes, Anaerotruncus*, *Intestinimonas*, and *Family XIII UCG 001* (*P* < 0.05, Kruskal–Wallis sum-rank test and Wilcoxon rank-sum test, Figure 2F, G). The relative abundance of *Bifidobacterium* in the intestine of the wild type and *ob/ob* groups was extremely low. After the intervention, there was a slight but not significant increase in the abundance of this genus, which was consistent with previous studies [11] (Figure S5).

### Metabolism-related signaling pathways changed in the cecum of leptin deficiency mice

To study the difference in the cecal transcriptome between wild type mice and genetically obese mice, RNA sequencing was performed. Comparative analysis of cecal transcriptomic profiles of *ob/ob* and wild type mice indicated that there were 1208 differentially expressed genes (DEGs) in the *ob/ob* mice compared to wild type (762 upregulated genes, including 242 genes with more than doubled expression levels; 446 downregulated genes, including 138 with more than halved expression levels, *P* < 0.05, Wald test, Figure S6). To further investigate the biological signaling pathways involved in the above DEGs, we used the Kyoto Encyclopedia of Genes and Genomes (KEGG) to do the enrichment analysis. We found that there were 232 signaling pathways involved in the DEGs between the *ob/ob* and wild type groups. Subsequently, according to the results of the correlation analysis, we obtained 50 candidate signaling pathways, which had strong correlations with the 5 metabolic parameters. These signaling pathways were mainly involved in certain KEGG categories, such as Environmental information processing, Organismal systems, and Metabolism. This result indicated that leptin gene deficiency in *ob/ob* mice affected some metabolism-related signaling pathways.

### The inulin-supplemented diet restores certain metabolism-related signaling pathways in *ob/ob* mice

**Figure 3:**
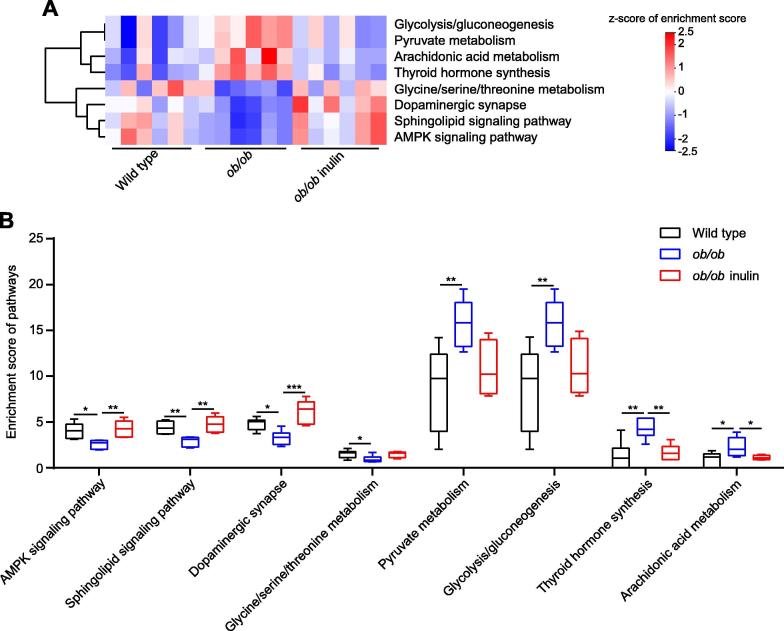
**Transcriptomic analyses of the cecal tissue** **A.** Heatmap of the expression values of 8 signaling pathways in each sample. The expression values of 18 samples are presented as the normalized z-score using the enrichment score of signaling pathways. **B.** Enrichment score of 8 signaling pathways. Data were analyzed using one-way ANOVA followed by the Tukey *post hoc* test for **B.***n* = 6 per group. ^*^P < 0.05; ^**^P < 0.01; ^***^P < 0.001.

We then investigated the effect of inulin treatment on the cecal transcriptome of obese mice. As the above results indicated that some metabolism-related signaling pathways of the cecum were changed in leptin gene deficiency mice, we continued to analyze the cecal transcriptome data after the inulin-supplemented diet. In total, there were 362 DEGs between *ob/ob* inulin and *ob/ob* (216 upregulated genes, including 42 genes with more than doubled expression; 146 downregulated genes, including 34 genes with more than halved expression levels, *P* < 0.05, Wald test, Figure S7). We found that 140 signaling pathways were involved in these DEGs. Using the same correlation analysis method as above, we selected 62 candidate signaling pathways. Finally, comparisons of the candidate pathways between *ob/ob vs*. wild type and *ob/ob* inulin *vs*. *ob/ob* identified 8 signaling pathways for which the enrichment score was changed due to leptin gene deficiency but restored by the inulin-supplemented diet (Figure 3A). In detail, the AMPK signaling pathway, Sphingolipid signaling pathway, Dopaminergic synapse, and Glycine/serine/threonine metabolism were downregulated, and in contrast, Pyruvate metabolism, Glycolysis/Gluconeogenesis, Arachidonic acid metabolism, and Thyroid hormone synthesis were elevated in the *ob/ob* group compared with the wild type group (*p* < 0.05, one-way ANOVA followed by the Tukey *post hoc* test, Figure 3B). Furthermore, the change in the enrichment score of these 8 signaling pathways was reversed after the inulin-supplemented diet (Table S1).

### Correlations among the bacteria, signaling pathways, and metabolic parameters

**Figure 4:**
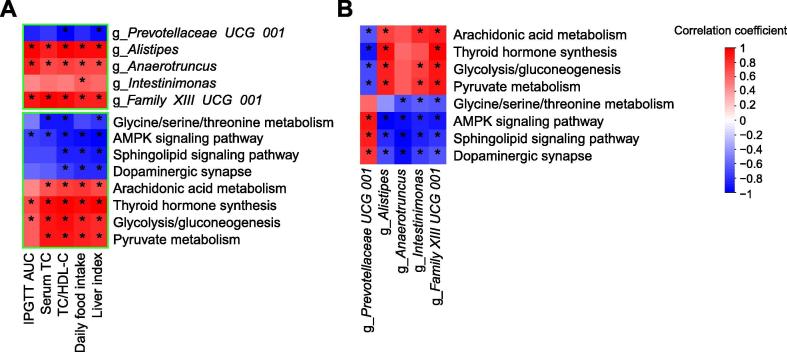
**Correlations among gut bacteria, signaling pathways, and metabolic parameters** **A.** Heatmap of the Spearman correlations between 5 genera, 8 signaling pathways and 5 metabolic parameters. **B.** Heatmap of the Spearman correlations between 5 genera and 8 signaling pathways. TC, total cholesterol; HDL-C, high-density lipoprotein cholesterol; AUC, area under the curve; IPGTT, intraperitoneal glucose tolerance test. *n* = 6 per group. ^*^P < 0.05.

Here, to further analyze the association between the cecal microbiota and the cecal transcriptome, we performed correlation analyses comparing the 5 bacterial genera and 8 signaling pathways with 5 metabolic parameters. These correlation analyses showed that *Prevotellaceae UCG 001* had a significant negative correlation with liver index and TC/HDL-C. However, *Alistipes*, *Anaerotruncus*, and *Family XIII UCG 001* were positively correlated with these parameters. Additionally, we found that the AMPK signaling pathway, Sphingolipid signaling pathway, Dopaminergic synapse, and Glycine/serine/threonine metabolism were negatively correlated with the 5 metabolic parameters, while Pyruvate metabolism, Glycolysis/Gluconeogenesis, Thyroid hormone synthesis, and Arachidonic acid metabolism had positive correlations with the metabolic parameters (Figure 4A).

Obviously, the gut microbiota could be affected by both leptin gene deficiency and inulin-supplemented diet. We performed correlation analyses between the 5 bacterial genera and 8 signaling pathways. The results showed that there was a significant positive correlation between the enrichment score of the AMPK signaling pathway and *Prevotellaceae UCG 001*, and the AMPK signaling pathway was negatively associated with the other 4 bacterial genera. However, the abundance of *Prevotellaceae UCG 001* had significant positive correlations with the enrichment scores of the AMPK signaling pathway, Sphingolipid signaling pathway, and Dopaminergic synapse. Conversely, *Alistipes* had significant positive correlations with Pyruvate metabolism, Glycolysis/Gluconeogenesis, Thyroid hormone synthesis, and Arachidonic acid metabolism, which were negatively correlated with the abundance of *Prevotellaceae UCG 001* (Figure 4B).

## Discussion

Disorder of the gut microbiota has been considered one of the reasons for metabolic disorders. Gut microbes can regulate gut micro-ecology through cell surface molecules and/or their metabolites, thereby affecting the host metabolic system, immune system, and nervous system [19], [22], [23], [24]. Although prebiotics can exert positive effects on the maintenance of host metabolic homeostasis, which are mainly mediated by gut microbiota [7], there are few reports on the effects of prebiotics on the intestinal gene expression [5], [11]. Genetically obese mice and lean mice have marked differences in several metabolic parameters and the gut microbiota [4]. Here, we reveal that the reason for the prebiotic inulin alleviating obesity-related glucose and lipid metabolism disorders in leptin gene deficiency mice may be closely related to the restoring of certain metabolism-related pathways.

In this study, after 4 weeks of the inulin-supplemented diet in *ob/ob* mice, significant alleviation of metabolic disorders was observed. Compared to the wild type group, the *ob/ob* group had a 71% higher body weight at 10 weeks of age, and their daily food intake increased by approximately 29%, which indicates that, due to the lack of functional leptin, the genetically obese mice lost their appetite control, but this status was improved after inulin treatment. We observed increases in serum TC, TC/HDL-C, and the AUC of IPGTT, which indicated that the glucose and lipid metabolism disorders were severe, as evidenced by the liver histopathology and liver index. The decreases in the above metabolic parameters after the inulin-supplemented diet suggest that the metabolic disorders in *ob/ob* mice were alleviated. Moreover, non-digestible fermentable dietary fibers can exert antidiabetic effects by increasing the secretion of GLP-1, which is secreted by intestinal epithelial cells, and this hormone can take part in maintaining blood glucose homeostasis [25]. We therefore measured the *Glp-1* mRNA level in the colon and found that this gene was significantly elevated after inulin treatment, suggesting that the maintenance of blood glucose homeostasis by inulin-supplemented diet was probably through the increased expression of *Glp-1* gene.

The abundance of the family Prevotellaceae (phylum Bacteroidetes) was increased by inulin, and *Prevotellaceae UCG 001* belonging to this family was enriched in the *ob/ob* inulin group. *Prevotella* possesses enzymes that can degrade cellulose and xylan [26]. As succinate-producing bacteria, *Prevotella* can participate in the degradation of inulin [27]. Dietary fiber facilitates the colonization of *Prevotella* in the gut, which in turn improves glucose metabolism [28]. A study utilizing wheat-type cereal products showed that cereals could alter the gut microbiota composition of infants, especially by increasing the family Prevotellaceae, which can degrade cellulose [29]. In a mouse model study, whole wheat oats altered the gut microbiota, increased the abundance of *Prevotella* and then enhanced insulin function and plasma lipid regulation [30]. In agreement with these observations, the abundance of *Prevotellaceae UCG 001* was negatively correlated with the blood glucose- and lipid-related parameters, especially TC/HDL-C and liver index. Therefore, the elevation in abundance of *Prevotellaceae UCG 001* might be closely related to the beneficial effect of the inulin-supplemented diet. Thus, we speculated that bacteria of this genus might affect the host glucose and lipid metabolism through the production of secondary metabolites, such as SCFAs.

In contrast with the lean mice, the abundance of *Alistipes* in the gut of the *ob/ob* mice was significantly increased but was reduced after the inulin-supplemented diet. Long-term ingestion of high-sugar foods can lead to enrichment of this genus in the human intestinal tract, as well as in animal experiments [31], [32]. The abundance of this genus, which contains pro-inflammatory bacteria, was significantly increased in the gut of obese individuals in Japan [33]. In this study, *Alistipes* was significantly positively correlated with serum TC, TC/HDL-C levels, and the AUC of IPGTT, which were identified as indicators of glucose and lipid metabolism disorders. Therefore, we speculated that when *Alistipes* was inhibited after the inulin-supplemented diet, its promotion of metabolic disorders decreased.

Arachidonic acid, an unsaturated fatty acid, is positively related to pro-inflammatory cytokines, but inversely correlated with *Akkermansia* and exacerbates non-alcoholic steatohepatitis (NASH) by promoting the growth of pro-inflammatory bacteria, enhancing the inflammatory response, reducing the abundance of butyrate-producing bacteria, and inducing insulin resistance [34], [35]. A high-fat diet can induce thyroid dysfunction in rodent models, and that the thyroid volume is significantly increased in obese individuals, in contrast to healthy ones [36], [37]. Here, the enrichment scores for Arachidonic acid metabolism and Thyroid hormone synthesis were restored to relatively lower levels after the inulin-supplemented diet.

Importantly, a significant increase in the enrichment score for the AMPK signaling pathway was exhibited after inulin supplementation. In vivo, the activation of AMPK signaling can stimulate glucose uptake, phosphorylation of acetyl coenzyme A carboxylase, hepatic glycolysis, lactate production and fatty acid oxidation, and correspondingly, it inhibits hepatic gluconeogenesis, cholesterol and fatty acid synthesis [38], [39]. AMPK has been well demonstrated that it can act as a crucial sensor in regulation of glycolipid metabolism. For example, AMPK influences energy intake, utilization, and storage in the skeletal muscle, heart, adipose tissue, liver, pancreatic beta cells, and brain by regulating dietary intake and substrate metabolism [20], [40]. The lactate and butyrate produced by certain intestinal bacteria can increase AMPK activity, which in turn increases fatty acid oxidation and energy expenditure [41]. Metformin and thiazolidinediones, widely used drugs for clinical treatment for diabetes, can promote fatty acid oxidation, and inhibit the activity of lipogenesis-related enzymes by activating AMPK in liver cells [42], [43]. Germ-free mice can resist the obesity induced by high-fat diets mainly by increasing AMPK phosphorylation in the liver and skeletal muscle. Phosphorylated AMPK can promote fatty acid oxidation in peripheral tissues [44]. Fat deposits in the liver and skeletal muscle are also regulated by intestinal bacteria through AMPK [45]. Obviously, AMPK plays a vital role in maintaining glycolipid homeostasis, but previous studies involving AMPK mainly focused on the alteration of this protein in the skeletal muscle and liver. In our study, in contrast to lean mice, the enrichment score of the AMPK signaling pathway in the cecum of *ob/ob* mice was reduced by 36%, and the *ob/ob* inulin mice had a 66% higher score than the *ob/ob* mice. The enrichment score of the AMPK signaling pathway was negatively correlated with liver index, daily food intake, serum TC, TC/HDL-C, and the AUC of IPGTT, but Glycolysis/Gluconeogenesis and Thyroid hormone synthesis showed positive correlations with these parameters. Furthermore, the results showed a high correlation between *Prevotellaceae UCG 001* and the enrichment score of the AMPK signaling pathway. In contrast, the abundance of *Alistipes* was significantly negatively correlated with this pathway. Interestingly, to date, correlations between the AMPK signaling pathway and these different bacteria have not been reported. Additionally, our findings emphasized the close interrelation between the cecal microbiota and the cecal transcriptome.

AMPK could be used as a potential target for the treatment of diabetes and obesity. AMPK activation in the liver can affect whole-body metabolism and lead to improvements in circulating levels of glucose and lipid [38], [46], and we found that this effect was not only present in the liver and skeletal muscle, but also in the cecum.

Compared with *ob/ob* group, the relative expression of colonic *Glp-1* mRNA was significantly increased in the *ob/ob* inulin group. As one of hormones produced by the gut, GLP-1 is a well-studied anorexigenic hormone. GLP-1 plays an important role in glucose homeostasis, gastrointestinal motility and appetite suppression [47], [48], [49]. SCFAs can stimulate the expression of GLP-1 from enteroendocrine L-cells, and the release of this hormone reduces food intake and maintains glucose homeostasis [50].

**Figure 5:**
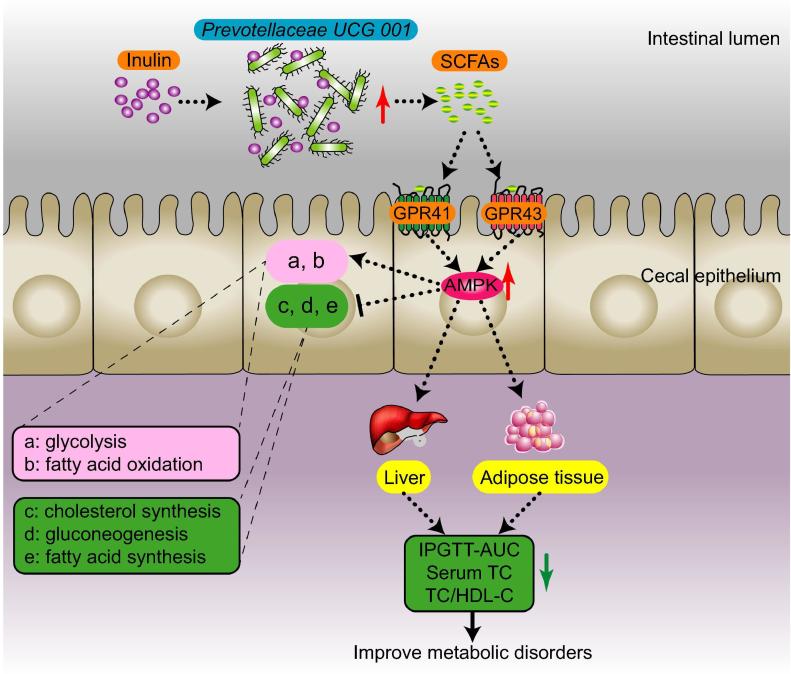
**Presumed mechanism by which inulin alleviated glucose and lipid metabolism disorders in *ob/ob* mice** The inulin-supplemented diet promotes the proliferation of *Prevotellaceae UCG 001* in the gut of *ob/ob* mice. This strain degrades inulin to produce SCFAs, which recognized GPR41/43 on the surface of intestinal epithelial cells, and lead to activation of the AMPK signaling pathway. Finally, this signal leads to changes in downstream metabolic functions. SCFAs, short-chain fatty acids; GPR41/43, G protein-coupled receptors 41/43; AMPK, adenosine monophosphate (AMP)-activated protein kinase; TC, total cholesterol; HDL-C, high-density lipoprotein cholesterol; AUC, area under the curve; IPGTT, intraperitoneal glucose tolerance test.

Overall, leptin gene deficiency led to metabolic disorders and inhibition of the AMPK signaling pathway in genetically obese mice. We speculated that the composition of cecal microbiota could be affected by inulin-supplemented diet. In particular, *Prevotellaceae UCG 001*, which produces SCFAs by degrading inulin, was increased. Then, SCFAs recognized and bound to GPR41/43 on the cell membrane of the cecal epithelium, triggering intracellular AMPK phosphorylation and the subsequent changes in downstream metabolic functions (such as promotion of fatty acid oxidation, glycolysis, and inhibition of hepatic fatty acid synthesis, cholesterol synthesis, and gluconeogenesis). Eventually, the host metabolic disorders were ameliorated (Figure 5).

## Conclusions

Prebiotic effect of inulin might be involving in the host transcriptome changing mediated by altering the gut microbiota. Based on the results of 16S rRNA gene amplicon analysis and transcriptomic analysis, we explored the complex interplay between the cecal microbiota and host gene expression profile. We first reported the effect of prebiotics on the cecal transcriptome in genetically obese mice. Giving inulin (10 g/kg/day) to *ob/ob* mice can alleviate glucose and lipid metabolism disorders, increase the abundance of *Prevotellaceae UCG 001*, and reduce the abundance of *Alistipes*. Moreover, we found that the AMPK signaling pathway in cecal tissue was upregulated after inulin supplementation. However, further studies are required to reveal the precise mechanism(s) behind these effects. Our results suggest that the AMPK signaling pathway not only plays a pivotal role in regulating metabolism in adipose tissue, liver, and skeletal muscle but also plays a vital role in the metabolic regulation mediated by gut microbiota. Furthermore, similar to the key role of the AMPK signaling pathway in the liver and skeletal muscle, the alteration of this pathway in the cecum may also play an indispensable role in the interaction between the cecal microbiota and the host metabolism system. Overall, signaling pathways related to energy metabolism in the gut are not negligible.

## Materials and methods

### Animals

Six-week-old male C57BL/6J mice were used in the experiment. C57BL/6J and *ob/ob* mice (C57BL/6J background; Huafukang Bio-Technique Co., Ltd, Beijing, China) were placed in a controlled environment (12 h sunlight cycle; lights off at 6 p.m.) with two mice in each cage given *ad libitum* access to feed and water. Upon delivery, the mice were acclimated for a period of 2 weeks, during which they were fed a normal chow diet (13.5% calories from fat; Vital River Laboratory Animal Technology Co., Ltd, Beijing, China). C57BL/6J mice were fed a normal chow diet (*n* = 6), while *ob/ob* mice were fed a normal chow diet supplemented (*n* = 6) or not (*n* = 6) with inulin (D908BA0044, Purity >90%, BBI Life Sciences Co., Ltd, Shanghai, China) at 10 g/kg/day through drinking water. The duration of the intervention was 4 weeks. Body weight was measured once weekly, and food and water intake were recorded daily.

*ob/ob* mice, which are deficient in the leptin gene (*Lep^ob^/Lep^ob^*), are one of the most common models for diabetes and obesity studies. There is a C→T mutation in C57BL/6J *ob/ob* mice that results in a change of an arginine at position 105 to a stop codon, which results in the synthesis of a truncated protein that is degraded in the adipocyte [51]. This gene deficiency can induce variations in key parameters such as glucose intolerance [52].

All animal experiments were approved by the Ethics Committee of the Institute of Microbiology, Chinese Academy of Sciences (IMCAS) and followed the Declaration of Helsinki (SQIMCAS2017009).

### IPGTT

For the glucose tolerance test, an IPGTT was carried out at the end (week 10) of the treatment. The mice were fasted for 12 h and then injected subcutaneously with glucose (2.0 g/kg body weight). The measurement of blood glucose with a blood glucose meter (Accu-Check, Roche, Switzerland) was conducted with tail bleeding at −30, 0, 15, 30, 60, 90, and 120 min before or after the intraperitoneal glucose load.

### Tissue sampling

At the end of the treatment period, the mice were anesthetized by an intraperitoneal injection of pelltobarbitalum natricum (80 mg/kg body weight). Serum samples were collected for further detection. Cervical dislocation was used to killed mice. Epididymal fat tissue, the liver, the spleen and intestinal segments (cecum and colon) were precisely dissected and weighed. Then, these tissues and serum samples were immediately submerged in liquid nitrogen and transferred to −80 °C for preservation.

### Histological analyses

Freshly isolated livers from all three groups were rapidly immersed in 4% formaldehyde for 24 h at ambient temperature. The tissues were then fixed in absolute ethyl alcohol for 24 h, and embedded in paraffin wax and cut into 5-μm-thick tissue slices. The slices were stained with hematoxylin/eosin (H&E) and detected by a DM2000 light microscope (Leica Microsystems GmbH, Wetzlar, Germany) at 100× magnification.

### Biochemical analyses

The whole blood was coagulated at room temperature for 30 min, and centrifuged at 4 °C and 3000×*g* for 10 min to collect serum. The serum was stored at −80 °C. The levels of serum TC, triglycerides (TG), HDL-C and low-density lipoprotein cholesterol (LDL-C) were measured using a commercial detection kit (NJJCBIO Co., Ltd, Nanjing, China) according to the kit instructions. Serum interleukin-1α (IL-1α), adiponectin (ADPN), and insulin were quantified using commercial enzyme-linked immunosorbent assay (ELISA) kits (NJJCBIO Co., Ltd, Nanjing, China) according to the kit instructions. Automated microplate reader was used to detect the absorbance values at 450 nm within 30 min.

### Microbiota analysis

After mice were dissected, the cecal contents were collected and stored at −80 °C. Microbial DNA was extracted from the cecal content using a TIANamp Stool DNA kit (TIANGEN Bio-Tech Co., Ltd, Beijing, China) according to the manufacturer’s protocol, including a bead-beating step. The V3-V4 region of the bacterial 16S ribosomal RNA gene was amplified by PCR (95 °C for 3 min, followed by 25 cycles at 95 °C for 30 s, 55 °C for 30 s, 72 °C for 30 s, and a final extension at 72 °C for 5 min) using primers F3 (CCTACGGGNBGCASCAG) and R4 (GTGCCAGCMGCCGCGGTAA). High-throughput sequencing was performed utilizing the Illumina HiSeq 2500 PE250 platform to detect the 16S rRNA amplicons according to standard protocols. Paired-end reads from sequencing were merged utilizing Fast Length Adjustment of SHort reads (FLASH) [53]. The fastq_quality_filter (-p 90 -q 25 -Q33) belonging to FASTX Toolkit 0.0.14 was used to filter low quality reads. USEARCH 64 bit v8.0.1517 was utilized to remove chimaeric reads. Normalizing the reads counts of each sample was based on the minimum size of samples by random subtraction. The UCLUST algorithm was used to align OTUs at 97% identity. The SILVA 16S rRNA database v128 was utilized to taxonomically classify OTUs. The Quantitative Insights Into Microbial Ecology (QIIME) was applied to calculate the α and β diversities, which were computed based on weighted and unweighted UniFrac distance matrices [54]. The significantly differential species between groups were generated by the LEfSe method [55].

### RNA-seq analysis

TRIzol reagent (Invitrogen, Thermo Scientific, MA, USA) was used to isolate total RNA of cecal samples following the instructions. Each sample required 4 μg of total RNA to construct a cDNA library. The library was constructed using the KAPA Stranded mRNA-Seq Kit for the Illumina platform (KAPA-BIO, Boston, USA) according to the manufacturer’s protocols. The library preparations were sequenced on an Illumina HiSeq PE150 platform.

The low quality reads and adaptor sequences were trimmed with Trimmomatic [56]. Clean reads were aligned to mm10 Hisat2 [57]. Gene expression levels were calculated by counting the overlap of reads on each gene with HT-seq [58] and normalized as reads per kilobase per million mapped reads (RPKM) with the gene annotation file from Ensembl (release 87) and the DESeq2 package in R [59]. In addition, the DESeq2 package was applied to calculate the differentially expressed genes (DEGs). Functional enrichment in Gene Ontology (GO) and KEGG was performed with the GOstats package [60]. The enrichment scores of pathways, which were used to compare the differences in pathways between two groups, were calculated by the RPKM values of the DEGs contained within each pathway.

### Availability of data

The datasets supporting the conclusions of this article are available in the NCBI repository (https://www.ncbi.nlm.nih.gov/); SRA accession SRP154971 for 16S rRNA gene amplicon sequencing data; SRA accession SRP155684 for RNA sequencing data.

### Real-time qPCR analysis

The FastQuant RT kit (TIANGEN BIO, Beijing, China) was applied to prepare cDNA according to the kit instructions. Real-time qPCR assays in triplicate were performed using the Applied Biosystems® 7500 Real-Time PCR System (Thermo Scientific, MA, USA) with KAPA SYBR FAST qPCR kit Master Mix (KAPA-BIO, Boston, USA). The expression of target genes in mice was normalized to that of the gene encoding glyceraldehyde-3-phosphate dehydrogenase (*GAPDH*) using the 2^-△△CT^ method. The qPCR primers, which were designed with PrimerBank [61], are listed in Table S2.

### Statistical analysis

Data represent mean ± standard error of the mean. For parametric variables, the unpaired two-tailed Student *t*-test was used to assess the differences in mean values between two groups. For three groups, statistical analysis was performed with ANOVA with Tukey *post hoc* test. For nonparametric variables, the statistical significance of the differences was evaluated by the Mann–Whitney test or Kruskal–Wallis test. For the IPGTT, two-way ANOVA was performed for the evolution of blood glucose levels with a *post hoc* test using Bonferroni method. *P* < 0.05 was considered statistically significant. GraphPad Prism 6 (GraphPad Software, San Diego, CA, USA) was used to do the statistical analyses.

## Authors’ contributions

BLZ and YFH conceived and designed the experiments. XFS, NL, and BXL performed the experiments. LZ, FL, and YNH performed the bioinformatics analysis. XFS, YQF, and YM performed the statistical analysis and interpreted the data. XFS, LZ, FL, YX, and JL wrote the initial manuscript. BLZ and YFH reviewed and edited the manuscript. All authors read and approved the final manuscript.

## Competing interests

The authors have declared no competing interests.

## Supplementary material

Supplementary Figure S1 **Initial body weight** Data are presented as mean ± SEM. Data were analyzed using one-way ANOVA followed by the Tukey post hoc test. *n* = 6 per group. ****P* < 0.001.

Supplementary Figure S2 **Body weight gain** Data are presented as mean ± SEM. Data were analyzed using one-way ANOVA followed by the Tukey post hoc test. *n* = 6 per group. ****P* < 0.001.

Supplementary Figure S3 **PCoA of the weighted UniFrac distances among the 3 groups**

Supplementary Figure S4 **Ratio of Firmicutes to Bacteroidetes** Data were analyzed using one-way ANOVA followed by the Tukey post hoc test. *n* = 6 per group.

Supplementary Figure S5 **Relative abundance of Bifidobacterium** Data were analyzed using one-way ANOVA followed by the Tukey post hoc test. *n* = 6 per group.

Supplementary Figure S6 **Volcano plot of the *ob/ob vs.* wild type mice** Red dots indicate differentially expressed genes (DEGs) in *ob/ob* in comparison with wild type mice. Green dots indicate wild type DEGs compared with *ob/ob*. Data were analyzed using Wald’s test. FC, Fold change.

Supplementary Figure S7 **Volcano plot of *ob/ob*-inulin *vs. ob/ob*** Red dots indicate *ob/ob*-inulin DEGs compared with *ob/ob* genes. Green dots indicate *ob/ob* DEGs compared with *ob/ob*-inulin genes. Data were analyzed using Wald’s test. FC, Fold change.

## References

[1] Bindels L.B., Delzenne N.M., Cani P.D., Walter J. (2015). Towards a more comprehensive concept for prebiotics. Nat Rev Gastroenterol Hepatol.

[2] Niness K.R. (1999). Inulin and oligofructose: what are they?. J Nutr.

[3] Everard A., Lazarevic V., Derrien M., Girard M., Muccioli G.G., Neyrinck A.M. (2011). Responses of gut microbiota and glucose and lipid metabolism to prebiotics in genetic obese and diet-induced leptin-resistant mice. Diabetes.

[4] Zhao L., Zhang F., Ding X., Wu G., Lam Y.Y., Wang X. (2018). Gut bacteria selectively promoted by dietary fibers alleviate type 2 diabetes. Science.

[5] Catry E., Bindels L.B., Tailleux A., Lestavel S., Neyrinck A.M., Goossens J.F. (2018). Targeting the gut microbiota with inulin-type fructans: preclinical demonstration of a novel approach in the management of endothelial dysfunction. Gut.

[6] Ghouri Y.A., Richards D.M., Rahimi E.F., Krill J.T., Jelinek K.A., DuPont A.W. (2014). Systematic review of randomized controlled trials of probiotics, prebiotics, and synbiotics in inflammatory bowel disease. Clin Exp Gastroenterol.

[7] Gibson G.R., Hutkins R., Sanders M.E., Prescott S.L., Reimer R.A., Salminen S.J. (2017). Expert consensus document: The International Scientific Association for Probiotics and Prebiotics (ISAPP) consensus statement on the definition and scope of prebiotics. Nat Rev Gastroenterol Hepatol.

[8] Dewulf E.M., Cani P.D., Claus S.P., Fuentes S., Puylaert P.G.B., Neyrinck A.M. (2013). Insight into the prebiotic concept: lessons from an exploratory, double blind intervention study with inulin-type fructans in obese women. Gut.

[9] Everard A., Belzer C., Geurts L., Ouwerkerk J.P., Druart C., Bindels L.B. (2013). Cross-talk between *Akkermansia muciniphila* and intestinal epithelium controls diet-induced obesity. Proc Natl Acad Sci U S A.

[10] Plovier H., Everard A., Druart C., Depommier C., Van Hul M., Geurts L. (2017). A purified membrane protein from *Akkermansia muciniphila* or the pasteurized bacterium improves metabolism in obese and diabetic mice. Nat Med.

[11] Zou J., Chassaing B., Singh V., Pellizzon M., Ricci M., Fythe M.D. (2018). Fiber-mediated nourishment of gut microbiota protects against diet-induced obesity by restoring IL-22-mediated colonic health. Cell Host Microbe.

[12] Roberfroid M., Gibson G.R., Hoyles L., McCartney A.L., Rastall R., Rowland I. (2010). Prebiotic effects: metabolic and health benefits. Br J Nutr.

[13] Kaji I., Karaki S., Kuwahara A. (2014). Short-chain fatty acid receptor and its contribution to glucagon-like peptide-1 release. Digestion.

[14] Murphy E.F., Cotter P.D., Healy S., Marques T.M., O'Sullivan O., Fouhy F. (2010). Composition and energy harvesting capacity of the gut microbiota: relationship to diet, obesity and time in mouse models. Gut.

[15] Ley R.E., Backhed F., Turnbaugh P., Lozupone C.A., Knight R.D., Gordon J.I. (2005). Obesity alters gut microbial ecology. Proc Natl Acad Sci U S A.

[16] Brown A.J., Goldsworthy S.M., Barnes A.A., Eilert M.M., Tcheang L., Daniels D. (2003). The Orphan G protein-coupled receptors *GPR41* and *GPR43* are activated by propionate and other short chain carboxylic acids. J Biol Chem.

[17] Cani P.D. (2018). Human gut microbiome: hopes, threats and promises. Gut.

[18] Cani P.D., Amar J., Iglesias M.A., Poggi M., Knauf C., Bastelica D. (2007). Metabolic endotoxemia initiates obesity and insulin resistance. Diabetes.

[19] Turnbaugh P.J., Ley R.E., Mahowald M.A., Magrini V., Mardis E.R., Gordon J.I. (2006). An obesity-associated gut microbiome with increased capacity for energy harvest. Nature.

[20] Kahn B.B., Alquier T., Carling D., Hardie D.G. (2005). AMP-activated protein kinase: ancient energy gauge provides clues to modern understanding of metabolism. Cell Metab.

[21] Hildebrandt M.A., Hoffmann C., Sherrill-Mix S.A., Keilbaugh S.A., Hamady M., Chen Y.Y. (2009). High-fat diet determines the composition of the murine gut microbiome independently of obesity. Gastroenterology.

[22] Turnbaugh P.J., Hamady M., Yatsunenko T., Cantarel B.L., Duncan A., Ley R.E. (2009). A core gut microbiome in obese and lean twins. Nature.

[23] Cani P.D., Bibiloni R., Knauf C., Waget A., Neyrinck A.M., Delzenne N.M. (2008). Changes in gut microbiota control metabolic endotoxemia-induced inflammation in high-fat diet-induced obesity and diabetes in mice. Diabetes.

[24] Wen L., Ley R.E., Volchkov P.Y., Stranges P.B., Avanesyan L., Stonebraker A.C. (2008). Innate immunity and intestinal microbiota in the development of Type 1 diabetes. Nature.

[25] Baggio L.L., Drucker D.J. (2007). Biology of incretins: GLP-1 and GIP. Gastroenterology.

[26] De Filippo C., Cavalieri D., Di Paola M., Ramazzotti M., Poullet J.B., Massart S. (2010). Impact of diet in shaping gut microbiota revealed by a comparative study in children from Europe and rural Africa. Proc Natl Acad Sci U S A.

[27] Nakayama J., Yamamoto A., Palermo-Conde L.A., Higashi K., Sonomoto K., Tan J. (2017). Impact of westernized diet on gut microbiota in children on leyte island. Front Microbiol.

[28] Kovatcheva-Datchary P., Nilsson A., Akrami R., Lee Y.S., De Vadder F., Arora T. (2015). Dietary fiber-induced improvement in glucose metabolism is associated with increased abundance of Prevotella. Cell Metab.

[29] Gamage H.K.A.H., Tetu S.G., Chong R.W.W., Ashton J., Packer N.H., Paulsen I.T. (2017). Cereal products derived from wheat, sorghum, rice and oats alter the infant gut microbiota in vitro. Sci Rep.

[30] Zhou A.L., Hergert N., Rompato G., Lefevre M. (2015). Whole grain oats improve insulin sensitivity and plasma cholesterol profile and modify gut microbiota composition in C57BL/6J mice. J Nutr.

[31] Yacoub R., Nugent M., Cai W., Nadkarni G.N., Chaves L.D., Abyad S. (2017). Advanced glycation end products dietary restriction effects on bacterial gut microbiota in peritoneal dialysis patients; a randomized open label controlled trial. PLoS One.

[32] Noble E.E., Hsu T.M., Jones R.B., Fodor A.A., Goran M.I., Kanoski S.E. (2017). Early-life sugar consumption affects the rat microbiome independently of obesity. J Nutr.

[33] Andoh A., Nishida A., Takahashi K., Inatomi O., Imaeda H., Bamba S. (2016). Comparison of the gut microbial community between obese and lean peoples using 16S gene sequencing in a Japanese population. J Clin Biochem Nutr.

[34] Ye J., Lv L., Wu W., Li Y., Shi D., Fang D. (2018). Butyrate protects mice against methionine-choline-deficient diet-induced non-alcoholic steatohepatitis by improving gut barrier function, attenuating inflammation and reducing endotoxin levels. Front Microbiol.

[35] Zhuang P., Shou Q., Lu Y., Wang G., Qiu J., Wang J. (2017). Arachidonic acid sex-dependently affects obesity through linking gut microbiota-driven inflammation to hypothalamus-adipose-liver axis. Biochim Biophys Acta.

[36] Yang Y., Zhang J., Wu G., Sun J., Wang Y., Guo H. (2018). Dietary methionine restriction regulated energy and protein homeostasis by improving thyroid function in high fat diet mice. Food Funct.

[37] Amouzegar A., Kazemian E., Abdi H., Mansournia M.A., Bakhtiyari M., Hosseini M.S. (2018). Association between thyroid function and development of different obesity phenotypes in euthyroid adults: A nine-year follow-up. Thyroid.

[38] Yamauchi T., Kamon J., Minokoshi Y., Ito Y., Waki H., Uchida S. (2002). Adiponectin stimulates glucose utilization and fatty-acid oxidation by activating AMP-activated protein kinase. Nat Med.

[39] Cool B., Zinker B., Chiou W., Kifle L., Cao N., Perham M. (2006). Identification and characterization of a small molecule AMPK activator that treats key components of type 2 diabetes and the metabolic syndrome. Cell Metab.

[40] Carvalho B.M., Saad M.J. (2013). Influence of gut microbiota on subclinical inflammation and insulin resistance. Mediators Inflamm.

[41] Gao Z., Yin J., Zhang J., Ward R.E., Martin R.J., Lefevre M. (2009). Butyrate improves insulin sensitivity and increases energy expenditure in mice. Diabetes.

[42] Fryer L.G., Parbu-Patel A., Carling D. (2002). The Anti-diabetic drugs rosiglitazone and metformin stimulate AMP-activated protein kinase through distinct signaling pathways. J Biol Chem.

[43] Zhou G., Myers R., Li Y., Chen Y., Shen X., Fenyk-Melody J. (2001). Role of AMP-activated protein kinase in mechanism of metformin action. J Clin Invest.

[44] Backhed F., Manchester J.K., Semenkovich C.F., Gordon J.I. (2007). Mechanisms underlying the resistance to diet-induced obesity in germ-free mice. Proc Natl Acad Sci U S A.

[45] John G.K., Wang L., Nanavati J., Twose C., Singh R., Mullin G. (2018). Dietary alteration of the gut microbiome and its impact on weight and fat mass: a systematic review and meta-analysis. Genes (Basel).

[46] Minokoshi Y., Kim Y.B., Peroni O.D., Fryer L.G., Muller C., Carling D. (2002). Leptin stimulates fatty-acid oxidation by activating AMP-activated protein kinase. Nature.

[47] Edholm T., Degerblad M., Gryback P., Hilsted L., Holst J.J., Jacobsson H. (2010). Differential incretin effects of GIP and GLP-1 on gastric emptying, appetite, and insulin-glucose homeostasis. Neurogastroenterol Motil.

[48] Hellstrom P.M., Naslund E., Edholm T., Schmidt P.T., Kristensen J., Theodorsson E. (2008). GLP-1 suppresses gastrointestinal motility and inhibits the migrating motor complex in healthy subjects and patients with irritable bowel syndrome. Neurogastroenterol Motil.

[49] Williams D.L., Baskin D.G., Schwartz M.W. (2009). Evidence that intestinal glucagon-like peptide-1 plays a physiological role in satiety. Endocrinology.

[50] Yadav H., Lee J.H., Lloyd J., Walter P., Rane S.G. (2013). Beneficial metabolic effects of a probiotic via butyrate-induced GLP-1 hormone secretion. J Biol Chem.

[51] Zhang Y., Proenca R., Maffei M., Barone M., Leopold L., Friedman J.M. (1994). Positional cloning of the mouse obese gene and its human homologue. Nature.

[52] Varga O., Harangi M., Olsson I.A., Hansen A.K. (2010). Contribution of animal models to the understanding of the metabolic syndrome: a systematic overview. Obes Rev.

[53] Magoc T., Salzberg S.L. (2011). FLASH: fast length adjustment of short reads to improve genome assemblies. Bioinformatics.

[54] Caporaso J.G., Kuczynski J., Stombaugh J., Bittinger K., Bushman F.D., Costello E.K. (2010). QIIME allows analysis of high-throughput community sequencing data. Nat Methods.

[55] Segata N., Izard J., Waldron L., Gevers D., Miropolsky L., Garrett W.S. (2011). Metagenomic biomarker discovery and explanation. Genome Biol.

[56] Bolger A.M., Lohse M., Usadel B. (2014). Trimmomatic: a flexible trimmer for Illumina sequence data. Bioinformatics.

[57] Kim D., Langmead B., Salzberg S.L. (2015). HISAT: a fast spliced aligner with low memory requirements. Nat Methods.

[58] Anders S., Pyl P.T., Huber W. (2015). HTSeq–a Python framework to work with high-throughput sequencing data. Bioinformatics.

[59] Love M.I., Huber W., Anders S. (2014). Moderated estimation of fold change and dispersion for RNA-seq data with DESeq2. Genome Biol.

[60] Falcon S., Gentleman R. (2007). Using GOstats to test gene lists for GO term association. Bioinformatics.

[61] Wang X., Spandidos A., Wang H., Seed B. (2012). PrimerBank: a PCR primer database for quantitative gene expression analysis, 2012 update. Nucleic Acids Res.

