# Supplementary figures and images for: Inulin Can Alleviate Metabolism Disorders in ob/ob Mice by Partially Restoring Leptin-related Pathways Mediated by Gut Microbiota
Source: Genomics Proteomics Bioinformatics. 2019 Apr 23;17(1):64–75. doi: 10.1016/j.gpb.2019.03.001 (PMC6520907; doi:10.1016/j.gpb.2019.03.001)

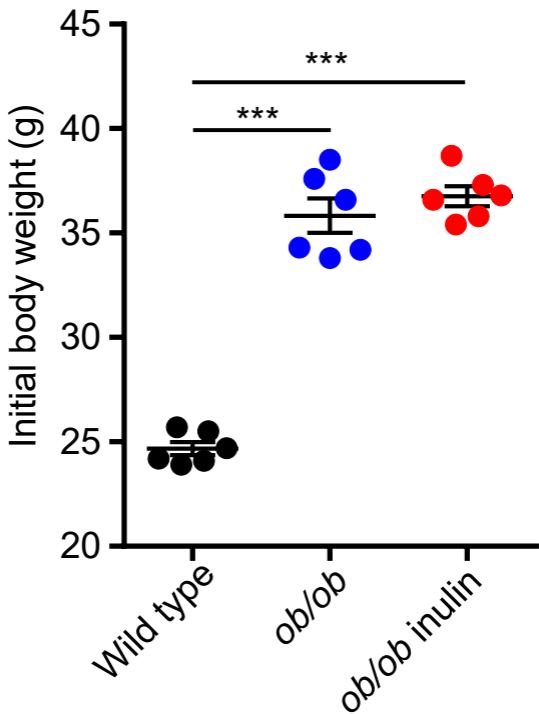

Supplement: Supplementary Figure S1 — Initial body weight Data are presented as mean ± SEM. Data were analyzed using one-way ANOVA followed by the Tukey post hoc test. n = 6 per group. ***P < 0.001. [file mmc1.pdf]

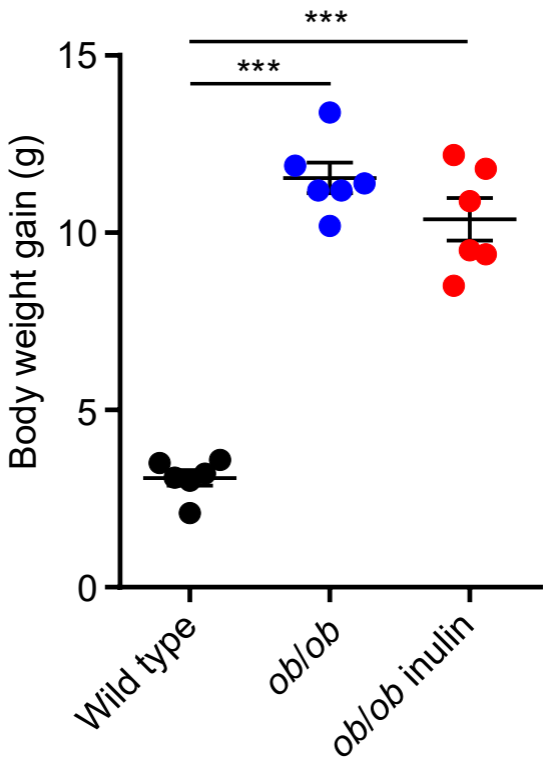

Supplement: Supplementary Figure S2 — Body weight gain Data are presented as mean ± SEM. Data were analyzed using one-way ANOVA followed by the Tukey post hoc test. n = 6 per group. ***P < 0.001. [file mmc2.pdf]

# PCoA-PC1 vs. PC3

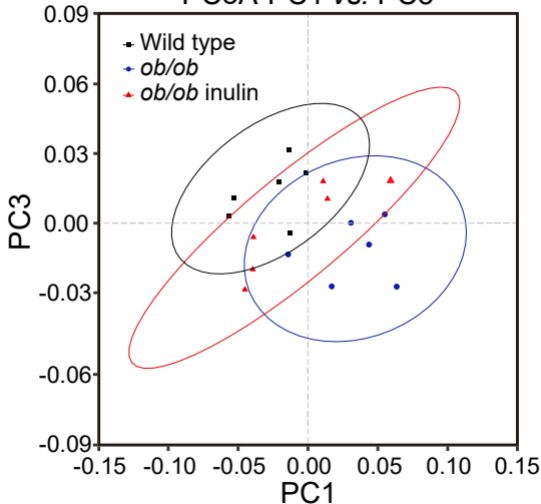

Supplement: Supplementary Figure S3 — PCoA of the weighted UniFrac distances among the 3 groups [file mmc3.pdf]

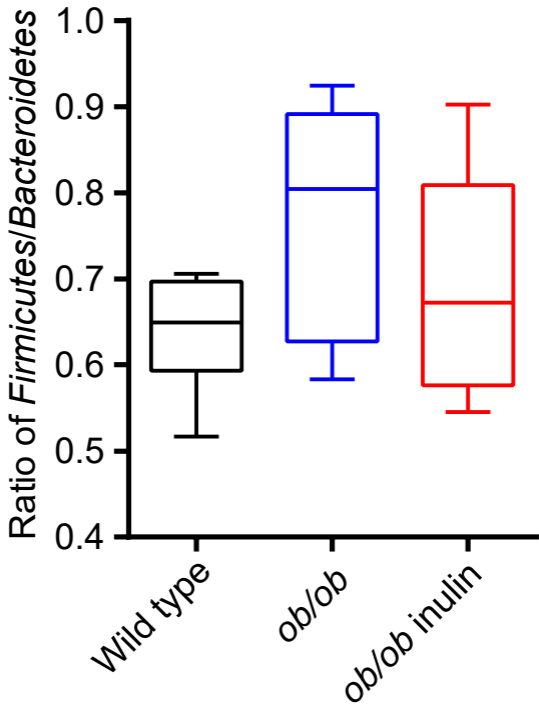

Supplement: Supplementary Figure S4 — Ratio of Firmicutes to Bacteroidetes Data were analyzed using one-way ANOVA followed by the Tukey post hoc test. n = 6 per group. [file mmc4.pdf]

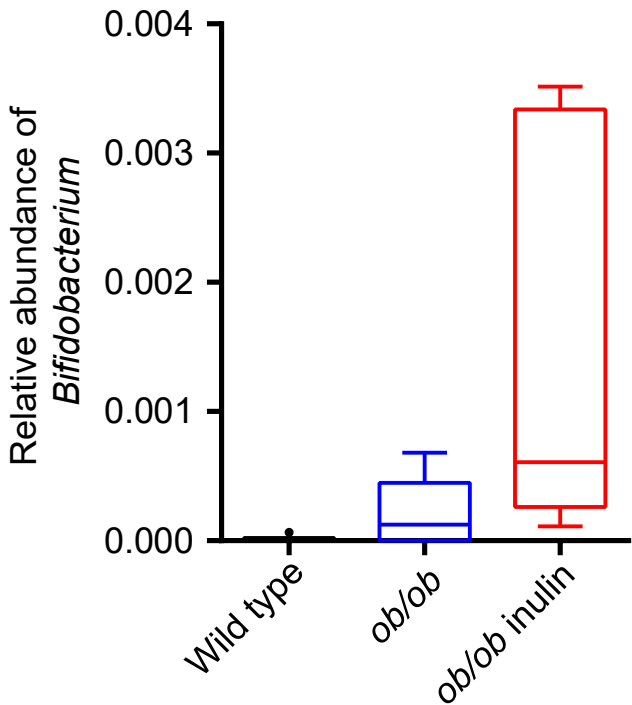

Supplement: Supplementary Figure S5 — Relative abundance of Bifidobacterium Data were analyzed using one-way ANOVA followed by the Tukey post hoc test. n = 6 per group. [file mmc5.pdf]

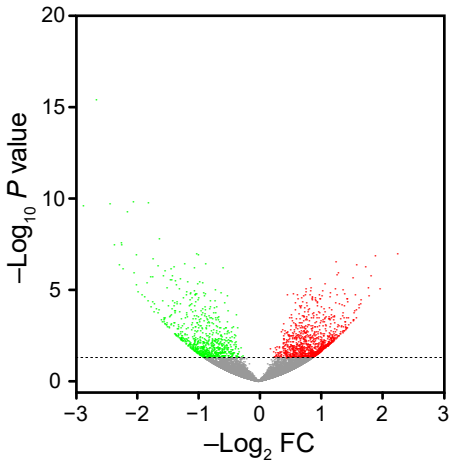

Supplement: Supplementary Figure S6 — Volcano plot of the ob/ob vs. wild type mice Red dots indicate differentially expressed genes (DEGs) in ob/ob in comparison with wild type mice. Green dots indicate wild type DEGs compared with ob/ob. Data were analyzed using Wald’s test. FC, Fold change. [file mmc6.pdf]

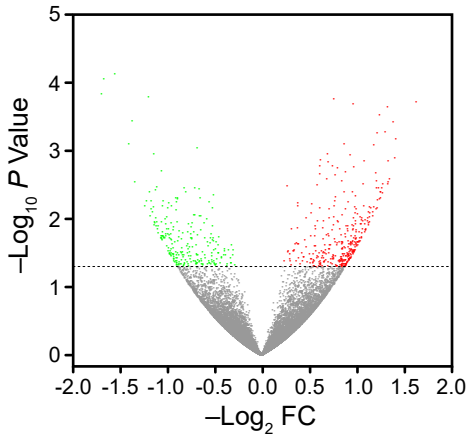

Supplement: Supplementary Figure S7 — Volcano plot of ob/ob-inulin vs. ob/ob Red dots indicate ob/ob-inulin DEGs compared with ob/ob genes. Green dots indicate ob/ob DEGs compared with ob/ob-inulin genes. Data were analyzed using Wald’s test. FC, Fold change. [file mmc7.pdf]
