# Supplementary material for: Inulin Can Alleviate Metabolism Disorders in ob/ob Mice by Partially Restoring Leptin-related Pathways Mediated by Gut Microbiota
Source: Genomics Proteomics Bioinformatics. 2019 Apr 23;17(1):64–75. doi: 10.1016/j.gpb.2019.03.001 (PMC6520907; doi:10.1016/j.gpb.2019.03.001)
Supplement: Supplementary Table S1 [file mmc8.docx]

**Table S1 Biological pathways identified by RNA sequencing analysis**

| Classification | Pathway | Enrichment Score | | | Change (%) |
| --- | --- | --- | --- | --- | --- |
|  |  | **Wild type** | ***ob/ob*** | ***ob/ob* inulin** | ***ob/ob* inulin *vs*. *ob/ob*** |
| Signal transduction | AMPK signaling pathway | 4.06±0.32 | 2.59±0.17 | 4.30±0.35 | 66^*^ |
| Signal transduction | Sphingolipid signaling pathway | 4.40±0.11 | 2.90±0.08 | 4.76±0.14 | 64^*^ |
| Amino acid metabolism | Glycine, serine and threonine metabolism | 1.52±0.07 | 0.94±0.06 | 1.48±0.05 | 57^*^ |
| Nervous system | Dopaminergic synapse | 4.79±0.10 | 3.30±0.12 | 6.16±0.19 | 87^*^ |
| Carbohydrate metabolism | Glycolysis/Gluconeogenesis | 8.70±1.73 | 15.81±0.97 | 10.93±1.08 | −45^*^ |
| Carbohydrate metabolism | Pyruvate metabolism | 8.69±1.73 | 15.80±0.97 | 10.83±1.07 | −46^*^ |
| Lipid metabolism | Arachidonic acid metabolism | 0.93±0.12 | 2.27±0.16 | 1.12±0.04 | −51^*^ |
| Endocrine system | Thyroid hormone synthesis | 1.19±0.23 | 4.28±0.16 | 1.69±0.13 | −61^*^ |

*Note*: Data are presented as the mean±SEM. **P* < 0.05 was considered by a two-tailed Student’s *t*-test with equal variance between the *ob/ob* and *ob/ob* inulin groups.
