# Supplementary material for: Inulin Can Alleviate Metabolism Disorders in ob/ob Mice by Partially Restoring Leptin-related Pathways Mediated by Gut Microbiota
Source: Genomics Proteomics Bioinformatics. 2019 Apr 23;17(1):64–75. doi: 10.1016/j.gpb.2019.03.001 (PMC6520907; doi:10.1016/j.gpb.2019.03.001)
Supplement: Supplementary Table S2 [file mmc9.docx]

**Table S2 Primers for real-time quantitative PCR**

| Genes | Forward primer (5′ to 3′) | Reverse primer (5′ to 3′) |
| --- | --- | --- |
| GAPDH | AGGTCGGTGTGAACGGATTTG | TGTAGACCATGTAGTTGAGGTCA |
| GLP-1 | AGGGACCTTTACCAGTGATGT | AATGGCGACTTCTTCTGGGAA |
| PPP2R3C | CTTCGGGAATCAGACCTGGAG | GCAGTGCAGACATAGAAGGAGT |
| Rab14 | GAGCGGTTACACGGAGCTAC | GTGAGATTCCTTGCGTCTGTC |
| Prkab1 | AGGCCCAAGATCCTCATGGA | GGGGGCTTTATCATTCGCTTC |

*Note*: GAPDH, glyceraldehyde-3-phosphate dehydrogenase; GLP-1, glucagon-like peptide-1; PPP2R3C, protein phosphatase 2, regulatory subunit B”, gamma; Rab14, member of the RAS oncogene family; Prkab1, protein kinase, AMP-activated, beta 1 non-catalytic subunit.
